# Metabolic Perspectives for Non-classical Congenital Adrenal Hyperplasia With Relation to the Classical Form of the Disease

**DOI:** 10.3389/fendo.2019.00681

**Published:** 2019-10-02

**Authors:** Djuro Macut, Vera Zdravković, Jelica Bjekić-Macut, George Mastorakos, Duarte Pignatelli

**Affiliations:** ^1^Clinic of Endocrinology, Diabetes and Metabolic Diseases, Clinical Center of Serbia, Faculty of Medicine, University of Belgrade, Belgrade, Serbia; ^2^Division of Endocrinology, University Children's Hospital, Faculty of Medicine, University of Belgrade, Belgrade, Serbia; ^3^Department of Endocrinology, UMC Bežanijska kosa, Faculty of Medicine, University of Belgrade, Belgrade, Serbia; ^4^Unit of Endocrine Diseases, Aretaieion Hospital, National and Kapodistrian University of Athens, Athens, Greece; ^5^Faculty of Medicine, Instituto de Patologia e Imunologia Molecular da Universidade do Porto/I3S Research Institute, Hospital S João, University of Porto, Porto, Portugal

**Keywords:** non-classical congenital adrenal hyperplasia, metabolism, obesity, cardiovascular risk, stature, osteoporosis, glucocorticoids, antiandrogens

## Abstract

Non-classical congenital adrenal hyperplasia (NC-CAH) represents mild form of CAH with the prevalence of 0. 6 to 9% in women with androgen excess. Clinical and hormonal findings in females with NC-CAH are overlapping with other hyperandrogenic entities such as polycystic ovary syndrome hence causing difficulties in diagnostic approach. Metabolic consequences in subjects with NC-CAH are relatively unknown. We are lacking longitudinal follow of these patients regarding natural course of the disease or the therapeutic effects of the different drug regiments. Patients with NC-CAH similarly to those with classical form are characterized with deteriorated cardiovascular risk factors that are probably translated into cardiometabolic diseases and events. An increased preponderance of obesity and insulin resistance in patients with NC-CAH begin at young age could result in increased rates of metabolic sequelae and cardiovascular disease later during adulthood in both sexes. On the other hand, growth disorder was not proven in patients with NC-CAH in comparison to CAH patients of both gender characterized with reduced final adult height. Similarly, decreased bone mineral density and osteoporosis are not constant findings in patients with NC-CAH and could depend on the sex, and type or dose of corticosteroids applied. It could be concluded that NC-CAH represent a particular form of CAH that is characterized with specificities in clinical presentation, diagnosis, therapeutic approach and metabolic outcomes.

## Introduction

The non-classical form of congenital adrenal hyperplasia (NC-CAH) was initially called late-onset as clinical presentation was observed in adolescents and adults. Presentation of NC-CAH is subtle and diagnosis requires implementation of different tests to exclude other problems. Moreover, clinical expression of NC-CAH is variable in patients carrying the same mutation. This suggests that additional factors may modify the clinical expression of the disease including age, steroid metabolic pathways, variation in androgen production, individual sensitivity to androgens, differences in skin sensitivity to androgens and possibly the existence of other genes modifying 21-hydroxylase activity (1, 2). In patients with NC-CAH predominant signs are those of androgen excess including premature pubarche, acne, hirsutism, polycystic ovary syndrome (PCOS) and subfertility (3).

NC-CAH is more frequently observed in females. The preponderance of NC-CAH in women with androgen excess, and according to ethnicity and genotype, is ranging from 0.6 to 9% (2). Males with NC-CAH are diagnosed significantly less often than females due to less frequently presented and recognizedsigns of androgen excess (4). One of the scarce and small studies showed in men with NC-CAH premature pubarche in 29% before 9 years of age (5). Gynecomastia has been reported as the presenting symptom in two male adolescents with non-classical congenital adrenal hyperplasia. Considering diagnosing NC-CAH, basal values of 17-OHP below 6 nmol/L were found in 2.1% of subjects with disease while concentrations of over 30 nmol/L after ACTH test is confirmatory for diagnosis (5).

As CAH is considered as disease spectrum, disease phenotype is ascertained by the less severe mutation with the highest residual enzymatic activity of CYP21A2 (6). The prevalence of classical CAH is from 1:10,000 to 1:20,000 births (7, 8) while in the non-classical CAH it is 1:1,000 births (9) and occurs in about 6% of hirsute women (10) (Supplementary Table 1).

Metabolic consequences in subjects with NC-CAH are relatively unknown and we are missing longitudinal follow up data of these patients regarding either the natural course of the disease or the outcomes of therapeutic regiments mainly based on glucocorticoids. Therefore, metabolic consequences in NC-CAH subjects could be analyzed from scarce clinical studies (11) or extrapolated from as well scarce clinical follow-up of patients with classical CAH.

## Clinical Characteristics in Different Age Periods

### Growth Differences

#### Patients With Non-classical CAH

Both boys and girls with NC-CAH could be characterized with fast linear growth, advanced bone age with consequent tall stature (4, 12). On the other hand, short stature was reported in subjects with NC-CAH as well particularly in patients on glucocorticoid therapy that commenced before the start of puberty. However, risk for short stature is relatively small with majority of children having almost normal final height (13).

#### Patients With Classical CAH

Birth length of salt wasting (SW) boys and girls at neonatal screening is above the average at birth while during infancy their height velocity declines by the age of 1.5 years in boys, and by the age of 3 years in girls (14). Patients with simple virilizing (SV) form of CAH have relative androgen insensitivity during the first year of life with consequent absence of increased height velocity (15). Before puberty, patients with SW are growing close to the reference values. True pubertal growth spurt is not noticeable in both forms and genders (16).

The first meta-analysis on the height outcome in classical CAH showed similar mean final height SD score (SDS) of −1.37 (~10 cm) in both genders compared to their target height (17). Studies on the outcomes in adults with classical CAH showed those on smaller glucocorticoid doses had slightly better SDS outcome of −1 below the target height. Patients with both CAH forms had reduced final adult height. However, women with simple virilizing form of CAH had shorter stature in comparison to those with salt wasting phenotype (18) (Supplementary Table 1).

### Sexual Maturation Characteristics

Howig et al. reported that the mean age for the onset of puberty was 9.8 years for girls with simple virilizing form, and 10.3 years for girls with SW CAH, and similar age at menarche of 13.3 and 13.7 years, respectively. In boys with SV form the onset of puberty was at 9.8, and 10.6 years for those with SW (19). Moreover, dissociation of pubarche and adrenarche was shown in classical CAH. Namely, in both boys and girls an earlier pubarche, gonadarche, and thelarche was confirmed with the absence of typical signs of adrenarche. Children having advanced skeletal maturation are at risk for early pubertal development (20). Elevated androgens may induce secondary central precocious puberty. High adrenal androgens resulting from non-suppression of HPA axis cause early puberty in both forms, more prevalent in SV than SW forms (21).

In those with NC-CAH pubertal delay, pubertal development progressed well after the initiation of glucocorticoid therapy with attainment of menarche and subsequently regular menstrual cycles (22) (Supplementary Table 1).

## Importance of Differential Diagnosis

### NC-CAH and Early Adrenarche/Pubarche

Adrenarche is characterized with elevated secretion of adrenal androgen precursors (AAPs), namely, dehydroepiandrosterone (DHEA) and DHEA-sulfate (DHEAS) that occurs at 5–8 years of age (23). Premature adrenarche (PA) and premature pubarche (PP) are defined as early appearance (before 9 years in boys and 8 years in girls) of axillary and pubic hair, adult type body odor or acne, with the absence of true central puberty. AAPs concentrations are above the prepubertal level with DHEAS > 1 μmol/l (termed as biochemical adrenarche) (24). The mechanisms of PA are not fully elucidated but the obesity and genetic factors seems to contribute in apparently multifactorial etiology (24).

Premature adrenarche is a benign condition but before the diagnosis can be accepted, differential diagnosis should consider defects of cortisol synthesis, androgen-producing tumors originated from adrenals or gonads, central precocious puberty, primary hypothyroidism, Cushing's syndrome, exposure to exogenous androgens, and most importantly NC-CAH (25).

The reported prevalence of CAH in PP patients differs from study populations (0–43% for all types of CAH) (26). Differential diagnosis between PA and NC-CAH, as well as other genetic reasons for adrenal hyperandrogenism, is not always obvious based on clinical examination. However, rapidly accelerating growth, remarkable androgenic signs and bone age advancement, and a positive family history are clues toward a genetic disorder (27).

### NC-CAH and PCOS

Clinical and hormonal findings that are overlapping with other hyperandrogenic entities such as PCOS may cause some difficulties in diagnostic approach in patient with suspected NC-CAH. Therefore, one should question what endocrine tests are appropriate to confirm or exclude NC-CAH, and if genetic analyses are indicated (5, 28, 29).

PCOS is considered frequent endocrine disease of women during reproductive period that is characterized with hyperandrogenism (30). As PCOS is a disease of exclusion, clinicians are obliged to perform necessary basal and functional endocrine testing to exclude other causes of androgen excess. Serum total testosterone determination is the main androgen determinant of PCOS (30). Approach to the hormonal analyses depends on the severity of hyperandrogenism. Determination of total testosterone is requested in patients with regular menstrual cycles and mild hirsutism (30). Determination of total testosterone together with FSH and LH is requested in patients with moderate hirsutism. In subjects with sudden development of hirsutism in adolescents and adults, and high concentrations of testosterone and androstenedione, an additional ACTH stimulation test with determination of 17-OHP together with CYP21A2 genotyping is requested (31).

Gonads could contribute to increase in 17-OHP as they also secrete this hormone. A hyperresponsiveness of 17-OHP to gonadotropin-releasing hormone agonist (GnRHa) is confirming ovarian hyperandrogenism. Hence, PCOS is considered as a result of ovarian thecal cell overactivity associated with 17,20-lyase activity down-regulation (32). As a consequence, hyperandrogenemia in PCOS could lead toward estrogen oversecretion. However, LH to estradiol ratio is kept normal in functional ovarian hyperandrogenism in the presence of elevated 17-OHP (33).

## Therapeutic Approach

### Therapy for NC-CAH

Concerning therapy for NC-CAH patients in contrast to classical CAH patients, adrenal replacement is not required. Therefore, pharmacological treatment is focused on the management of the signs of androgen excess. Use of glucocorticosteroid (GCS) therapy should be reserved for special outcomes such as restauration of fertility. When talking on the long-term outcomes of androgen excess including menstrual cycle derangement, first line options are antiandrogens and oral contraceptives (34) (Supplementary Table 1).

### Therapy for Classical CAH

#### Glucocorticoids and All Forms of CAH

Longitudinal growth and bone age development are the most important clinical parameters for monitoring adequate glucocorticoid replacement in children with CAH (35). Serum levels of 17-OHP, androstenedione and testosterone need to be monitored frequently and the hydrocortisone dose adjusted to maintain treatment efficacy. The levels of androstenedione and testosterone are more important in monitoring the glucocorticoid dosage than 17-OHP that should be kept a little above the normal range.

Analysis of growth in CAH patients reveal that the two most rapid phases of growth (first year of life and pubertal growth spurt) are vulnerable ones, during which glucocorticoid overtreatment should be avoided (14, 19). During infancy hydrocortisone dose should be decreased in patients with slower growth velocity, but strict hydrocortisone dose adjustments are necessary during childhood to avoid accelerated growth and advanced skeletal maturation (16). Pharmacokinetics of free cortisol showed shorter half-life in pubertal girls than in pubertal boys (36). Consequently, female patients at puberty need more frequent hydrocortisone replacement regimen. Besides decreased compliance of adolescents with CAH, use of oral contraceptive pills, aromatase inhibitors, thyroxine, or ingestion of hydrocortisone with food could influence cortisol dynamics and altering hydrocortisone dosing (37).

Hydrocortisone three times per day is the mostly prescribed regimen by pediatricians (38). Prednisolone or dexamethasone could be used in children with late puberty and in adults, but are avoided in childhood due to the potent effect on growth. It was observed several rises in cortisol concentrations during the day in children on hydrocortisone therapy. These phased are interchanged with periods of hypocortisolemia between the doses, especially during the night. Reduction of glucocorticoid dose during puberty is of clinical importance as pubertal height gain represent a predictor of final height. On the other hand, low glucocorticoid dose with concomitant hyperandrogenism can lead to premature epiphyseal closure and short stature (17). Other treatment options to improve final height and metabolic outcome might include subcutaneous hydrocortisone infusion through a pump, as well as plenadren (modified-release hydrocortisone) and chronocort (modified-release hydrocortisone formula, under development) that have a delayed and sustained absorption profile (38) (Supplementary Table 1).

#### Mineralocorticoids

Endocrine Society (USA) recommended fludrocortisone (0.05–0.2 mg once or twice per day) to be used in all patients with classical CAH (13). Fludrocortisone is used more frequently and in higher doses in patients with more severe genotypes. Overtreatment, defined by renin concentrations either in the lower reference range or suppressed, was only rarely observed throughout the different mutation groups (22) (Supplementary Table 1).

## Metabolic Outcomes of NC-CAH

### Obesity and Cardiometabolic Risk During Life

#### Non-classical CAH

One third of untreated NC-CAH women have insulin resistance (11, 39). In children with both classical CAH and NC-CAH prevalence of obesity is approximately 35% and exceeds obesity rates in children and adolescent in the general population (18). Obese children with either forms of CAH are hyperinsulinemic and hyperleptinemic but predominantly in those with classical form of CAH (18, 40). However, Saygili et al. did not show difference in leptin concentrations in hyperinsulinemic and hyperandrogenemic adult women with NC-CAH in comparison to controls (39). Moreover, an association of leptin levels with hyperinsulinemia and hyperandrogenism in women with NC-CAH was not established with conclusion that potential relation of leptin with hyperinsulinemia and reproduction in NC-CAH patients need further investigation (39).

We are lacking clinical studies in NC-CAH children regarding obesity, metabolic syndrome and their consequences (41). Concerning adult NC-CAH patients, it was recently shown an increased risk of metabolic and cardiovascular morbidities in both males and females (42).

Adrenal androgen secretion is increased in NC-CAH in the presence of normal levels of ACTH in the majority of subjects (43). Moreover, NC-CAH patients have normal levels of DHEAS while androstenedione and testosterone are similarly elevated to the levels in PCOS women (43, 44). As mentioned previously, altered enzyme kinetics due to CYP21A2 missense mutations is associated with adrenal androgen oversecretion in NC-CAH (2). Another mechanisms contributing to hyperandrogenism in NC-CAH includes ovarian dysfunction and peripheral synthesis of androgens from steroid precursors (41). As a consequence, use of glucocorticoids around puberty in hyperandrogenic state could favor abdominal visceral adiposity, insulin resistance with concomitant metabolic derangement and further exacerbation of androgen production (45). It was recently proposed a backdoor pathway for hyperandrogenism of NC-CAH with transformation of 17-OHP and progesterone into more potent androgens such as dihydrotestosterone (46) (Supplementary Table 1).

#### Classical CAH

It was observed an existence of higher rates of obesity and insulin resistance in adult patients with CAH (22). Hyperandrogenism was suggested as an intrinsic hormonal imbalance in classical CAH. Life-long glucocorticoid treatments are conferring to the increased risk for obesity and cardiovascular disease (CVD). Moreover, higher doses of glucocorticoids were associated with obesity in adults irrespectively on their family predisposition to obesity (18).

Leptin and other adipokines are elevated in almost all ages of patients with classical CAH, and characterized with abdominal obesity, consequent changes in food consumption, insulin sensitivity, and energy homeostasis. Therefore, it seems that adipokines are involved in the pathogenesis of obesity in patients with CAH (47). The increase in amount of fat commenced during childhood, it is existing even in children adequately treated, and was found in young adults with CAH as well (48). However, we are lacking information on the metabolic activity of abdominal adipose tissue in CAH patients. Recently, Kim et al. (47) showed that CAH adolescents and young adults have increased abdominal adiposity, with a higher proportion of pro inflammatory visceral adipose tissue (VAT) than subcutaneous adipose tissue. This places CAH patients at even greater risk for harmful metabolic sequelae from obesity linked to CVD risk. Moreover, strong correlation was obtained between VAT and adipokines or inflammatory markers (i.e., leptin, PAI-1, and hs-CRP). These findings implicated on the association of insulin resistance and metabolic syndrome in young patients with CAH (49). Consequently, adolescents and young adults with CAH express similar low-grade inflammation as obese individuals without CAH (49).

Clinical studies based on dual x-ray absorptiometry (DXA) showed that either males or females with classic CAH exhibit higher total fat mass and reduced lean body mass than controls. In respect to therapy used, a week correlation was found for cumulative glucocorticoid dose and total fat mass in females only (50). However, there was no observed differences between males and females for body composition or obesity in either classical CAH or NC-CAH (18, 40, 47, 51) (Supplementary Table 1).

#### Impact of Therapies on the Disease Prevalence

In respect to the therapy used, different studies analyzing patients with classical CAH showed variation in prevalence of obesity from 16.8 to 35% on hydrocortisone doses ranging from 13.3 to 15 mg/m^2^/day while only one study reported prevalence of obesity of 60.7% on the dose of 19.5 mg/m^2^/day (50). As meta-analyses was not performed yet, it could not be suggested a linear correlation between the prevalence of obesity and increasing doses of hydrocortisone as a frequently used therapeutic modality.

Many studies analyzing patients with CAH indicate an increased prevalence of insulin resistance but very few indicated an increased prevalence of diabetes including gestational diabetes. This could be partly explained with CAH studies analyzing patients younger than 50 as age group that is not typical for development of diabetes (4). In respect to glucocorticosteroids used, a few reports on classical CAH patients are giving opposite results with elevated insulin resistance index (HOMA-IR) in 18 and 44.4% on hydrocortisone doses of 19.5 and 11.2 mg/m^2^/day, respectively (50).

### Androgen Excess and Cardiovascular Risk

Classical CAH as hyperandrogenic state is characterized in women with increased cardiovascular risk (52). Either low or high testosterone levels have an increased risk for CVD independently of age, adiposity, ovarian function, and smoking (53). Traditional and non-traditional cardiovascular risk markers as well as their functional and morphological effects recognized throughout the surrogate indices, imply on the existence of increased cardiovascular risk and subclinical CVD in various types of women with androgen excess (52). Study by Falhammar et al. (54) on small number of patients analyzed cardiovascular and metabolic risk profiles in adult CAH males on lifelong glucocorticoid treatment. The authors showed that younger CAH males did not differ from age-matched controls while risk increased in subjects older than 30 years (54). However, we are lacking data on the frequency of established cardiovascular disease and mortality. It was shown that stroke is common in women with NC-CAH (odds ratio 5.8) while acute coronary syndrome is characteristic for males with classical CAH (odds ratio 9.9) (42). Hazard ratio of death was higher for females, and with 32% caused by cardiovascular diseases (55).

Higher concentrations of testosterone and DHEA in post-menopausal women are associated with the progression of atherosclerosis and development of hypertension (56). It was recently suggested an association of hyperandrogenism with the occurrence of inflammation and oxidative stress at the level of vascular endothelium, and concomitant renal reabsorption of sodium and water (56). Androgens have an adverse effects on insulin sensitivity, visceral adiposity and lipolysis, low-density-lipoprotein cholesterol (LDL-C) clearance, and high-density-lipoprotein cholesterol (HDL-C) concentrations (57). Moreover, androgen excess is deteriorating lipid profile making it more atherogenic through lower HDL-C, increased LDL-C level as a consequence of blunted LDL-receptor activity, or by enhanced lipoprotein lipase activity (57).

CAH is characterized by an existence of cardiovascular risk factors including dyslipidemia, obesity and insulin resistance, as well as cardiovascular outcomes as hypertension (58). There is a similarity in respect to the presence of cardiovascular risk factors in CAH patients and PCOS patients. Glucocorticoid used in high doses for the androgen suppression could induce Cushing syndrome characteristics with further aggravation of existing cardiovascular risk factors (59). Some data in children's populations showed decreased HDL-C in ~10% of both classic CAH and NC-CAH subjects (18). In another study, an association of androgen concentrations with dyslipidemia, obesity, and IR was shown in CAH patients (58) as well as elevated triglyceride concentrations confirmed in children with classical CAH on prednisone therapy (60). Moreover, prepubertal children with classical CAH could have elevated leptin and insulin levels constituting a group with increased life-long cardiovascular risk (61).

Hypertension is frequent finding in patients with classical CAH. It could be a consequence of the disease or the effect of therapy. Moreover, hypertension is considered the main cause of cardiovascular morbidity in young patients with CAH (62). In respect to the activity of the disease, elevated blood pressure was more likely diagnosed all patients with classical CAH than in NC-CAH ones, and associated with children of younger age and adult male gender, respectively (18). Moreover, deranged systolic blood pressure in children with classical CAH was characterized with higher daytime values and the absence of the nocturnal descent (63). Monitoring of blood pressure in children is of importance. Namely, it was observed an increased hypertension rate in young patients with classical CAH on fludrocortisone therapy (64). Available studies on CAH patients of different age showed similar prevalence of hypertension between males and females with CAH (18, 40, 63) with few exceptions showing more deteriorated BP in younger females likely to be related to androgen excess (50).

Carotid intima-media thickness (CIMT) is used as surrogate marker of arterial damage. It was shown that CIMT was increased in CAH patients including different age groups and independently of androgen levels, insulin levels or glucocorticoid treatment (65). Moreover, when comparing adolescents with classical CAH and NC-CAH, even those with NC-CAH may be at higher risk of having increased CIMT, possibly related to intermittent iatrogenic hypercortisolism and secondary insulin resistance (66). In respect to gender differences, there was no association between CIMT and androstenedione and 17OH progesterone in exposed females with classical CAH (51) (Supplementary Table 1).

### Bone Mineral Density and Risk for Osteoporosis

Adult patients with classical CAH are shorter than average individuals in the general population by ~10 cm (17). This is caused by the parallel effects of earlier exposure to androgens causing accelerated growth rate in childhood and premature epiphyseal closure in the long bones, and suppressive effects of excessive GCS doses on the secretion of growth hormone on the other.

The age and duration-related data on the assessment of bone mineral density (BMD) in patients with CAH are varying. While some authors showed lower BMD in adults with classical CAH and NC-CAH (18) others showed differences in BMD in relation only to glucocorticoid use (67). Curiously, while GCS therapy in children did not show a decrease in BMD, significantly lower BMD was demonstrated in adult patients with CAH on GCS treatment, and specially in those having salt-wasting CAH managed with highest doses of GCS (68, 69). In respect to the drug used, lower BMD values are found in patients managed with longer-acting GCS compared to those managed with hydrocortisone (70). An additional factor influencing decrease in BMD could be the reduced levels of DHEA and DHEAS that was shown in post-menopausal women treated with long-acting GCS (68).

Apart from the assessment of height and BMD using dual energy X-ray absorptiometry, there is not an established role for the bone turnover biochemical markers in patients with CAH (69).

Use of glucocorticoids could result in long-term complications including osteoporosis and fractures (4). In respect to the CAH variant, while some authors reported normal or even better BMD in different age-groups of patients with NC-CAH in comparison to classical CAH (71, 72), other authors found similar frequency of osteoporosis in NCAH in comparison to classical CAH (18).

Osteoporosis could be expected in adults with classical CAH on chronic glucocorticoid therapy. GCS therapy is influencing the activity of osteoblasts with consequent decreased in BMD (67). In line with this is low activity of the markers of bone formation such as osteocalcin, in adults with CAH (73). Apart from the more pronounced decrease in BMD after the age of 30, an increased rate of osteoporotic fractures was shown in women with classic CAH treated with GCS (71). Moreover, males older than 30 years had lower BMD in all measured sites similarly to women of the same age (67, 71). However, in spite of lower BMD and osteocalcin concentrations in males on prednisolone therapy in comparison to the ones hydrocortisone treatment, they did not have higher frequency of fractures (67). Taking into consideration all the aspects of GCS treatment on BMD, and in spite of decreased bone turnover markers, most patients with CAH have normal BMD. Preservation of bone integrity over GCS treatment could be explained with the commonly increased body mass index, which is protective to bones, as well as with the anabolic effects of androgens in both males and females (73). However, the most likely explanation for the preserved BMD could rely in the positive net effect of the GCS type and dose adjustment during patient's follow-up (Supplementary Table 1).

## Conclusion

Patients with NC-CAH are prone to develop metabolic consequences. Those patients are with higher rates of obesity and insulin resistance, as well as with increased rates of metabolic sequelae and cardiovascular disease during an adult period in both males and females. These patients could be at higher risk of having increased arterial intima-media thickness, possibly related to intermittent iatrogenic hypercortisolism and secondary insulin resistance. Decreased BMD and osteoporosis is not a constant finding in these patients and could depend on the sex, and type or dose of glucocorticosteroids applied.

## Author Contributions

The present work was designed by DM, VZ, JB-M, GM, and DP. The initial manuscript draft was prepared by DM and subsequently revised by VZ, J-BM, GM, and DP. All the authors approved the final submitted version.

### Conflict of Interest

The authors declare that the research was conducted in the absence of any commercial or financial relationships that could be construed as a potential conflict of interest.

## References

[1] Bidet M Bellanne-Chantelot C Galand-Portier MB Tardy V Billaud L Laborde K. Clinical and molecular characterization of a cohort of 161 unrelated women with nonclassical congenital adrenal hyperplasia due to 21-hydroxylase deficiency and 330 family members. J Clin Endocrinol Metab. (2009) 94:1570–8. 10.1210/jc.2008-1582 19208730

[2] Witchel SF Azziz R. Nonclassic congenital adrenal hyperplasia. Int J Pediatr Endocrinol. (2010) 2010:625105. 10.1186/1687-9856-2010-625105 20671993 PMC2910408

[3] Marino R Ramirez P Galeano J Perez Garrido N Rocco C Ciaccio M. Steroid 21-hydroxylase gene mutational spectrum in 454 Argentinean patients: genotype-phenotype correlation in a large cohort of patients with congenital adrenal hyperplasia. Clin Endocrinol. (2011) 75:427–35. 10.1111/j.1365-2265.2011.04123.x 21609351

[4] Nordenstrom A Falhammar H Management of endocrine disease: diagnosis and management of the patient with non-classic CAH due to 21-hydroxylase deficiency. Eur J Endocrinol. (2019) 180:R127–45. 10.1530/EJE-18-0712 30566904

[5] Livadas S Dracopoulou M Dastamani A Sertedaki A Maniati-Christidi M Magiakou AM. The spectrum of clinical, hormonal and molecular findings in 280 individuals with nonclassical congenital adrenal hyperplasia caused by mutations of the CYP21A2 gene. Clin Endocrinol. (2015) 82:543–9. 10.1111/cen.12543 25041270

[6] White PC Speiser PW. Congenital adrenal hyperplasia due to 21-hydroxylase deficiency. Endocr Rev. (2000) 21:245–91. 10.1210/er.21.3.245 10857554

[7] Nordenstrom A Ahmed S Jones J Coleman M Price DA Clayton PE. Female preponderance in congenital adrenal hyperplasia due to CYP21 deficiency in England: implications for neonatal screening. Horm Res. (2005) 63:22–8. 10.1159/000082896 15627780

[8] van der Kamp HJ Wit JM. Neonatal screening for congenital adrenal hyperplasia. Eur J Endocrinol. (2004) 151(Suppl. 3):U71–5. 10.1530/eje.0.151u071 15554889

[9] Speiser PW Dupont B Rubinstein P Piazza A Kastelan A New MI. High frequency of nonclassical steroid 21-hydroxylase deficiency. Am J Hum Genet. (1985) 37:650–67. 9556656 PMC1684620

[10] Kuttenn F Couillin P Girard F Billaud L Vincens M Boucekkine C. Late-onset adrenal hyperplasia in hirsutism. N Engl J Med. (1985) 313:224–31. 10.1056/NEJM198507253130404 2989686

[11] Pall M Azziz R Beires J Pignatelli D. The phenotype of hirsute women: a comparison of polycystic ovary syndrome and 21-hydroxylase-deficient nonclassic adrenal hyperplasia. Fertil Steril. (2010) 94:684–9. 10.1016/j.fertnstert.2009.06.025 19726039

[12] Oberfield SE Sopher AB Gerken AT. Approach to the girl with early onset of pubic hair. J Clin Endocrinol Metab. (2011) 96:1610–22. 10.1210/jc.2011-0225 21602454 PMC3100750

[13] Speiser PW Azziz R Baskin LS Ghizzoni L Hensle TW Merke DP. Congenital adrenal hyperplasia due to steroid 21-hydroxylase deficiency: an Endocrine Society clinical practice guideline. J Clin Endocrinol Metab. (2010) 95:4133–60. 10.1210/jc.2009-2631 20823466 PMC2936060

[14] Bonfig W Schmidt H Schwarz HP. Growth patterns in the first three years of life in children with classical congenital adrenal hyperplasia diagnosed by newborn screening and treated with low doses of hydrocortisone. Horm Res Paediatr. (2011) 75:32–7. 10.1159/000316973 20714115

[15] Bonfig W Schwarz HP. Growth pattern of untreated boys with simple virilizing congenital adrenal hyperplasia indicates relative androgen insensitivity during the first six months of life. Horm Res Paediatr. (2011) 75:264–8. 10.1159/000322580 21196707

[16] Bonfig W. Growth and development in children with classic congenital adrenal hyperplasia. Curr Opin Endocrinol Diabetes Obes. (2017) 24:39–42. 10.1097/MED.0000000000000308 27898585

[17] Eugster EA Dimeglio LA Wright JC Freidenberg GR Seshadri R Pescovitz OH. Height outcome in congenital adrenal hyperplasia caused by 21-hydroxylase deficiency: a meta-analysis. J Pediatr. (2001) 138:26–32. 10.1067/mpd.2001.110527 11148508

[18] Finkielstain GP Kim MS Sinaii N Nishitani M Van Ryzin C Hill SC. Clinical characteristics of a cohort of 244 patients with congenital adrenal hyperplasia. J Clin Endocrinol Metab. (2012) 97:4429–38. 10.1210/jc.2012-2102 22990093 PMC3513542

[19] Bonfig W Pozza SB Schmidt H Pagel P Knorr D Schwarz HP. Hydrocortisone dosing during puberty in patients with classical congenital adrenal hyperplasia: an evidence-based recommendation. J Clin Endocrinol Metab. (2009) 94:3882–8. 10.1210/jc.2009-0942 19622620

[20] Volkl TM Ohl L Rauh M Schofl C Dorr HG. Adrenarche and puberty in children with classic congenital adrenal hyperplasia due to 21-hydroxylase deficiency. Horm Res Paediatr. (2011) 76:400–10. 10.1159/000333696 22123283

[21] Dacou-Voutetakis C Karidis N. Congenital adrenal hyperplasia complicated by central precocious puberty: treatment with LHRH-agonist analogue. Ann N Y Acad Sci. (1993) 687:250–4. 10.1111/j.1749-6632.1993.tb43873.x 8323180

[22] Krone N Rose IT Willis DS Hodson J Wild SH Doherty EJ. Genotype-phenotype correlation in 153 adult patients with congenital adrenal hyperplasia due to 21-hydroxylase deficiency: analysis of the United Kingdom Congenital adrenal Hyperplasia Adult Study Executive (CaHASE) cohort. J Clin Endocrinol Metab. (2013) 98:E346–54. 10.1210/jc.2012-3343 23337727 PMC3651585

[23] Idkowiak J Lavery GG Dhir V Barrett TG Stewart PM Krone N. Premature adrenarche: novel lessons from early onset androgen excess. Eur J Endocrinol. (2011) 165:189–207. 10.1530/EJE-11-0223 21622478

[24] Voutilainen R Jaaskelainen J. Premature adrenarche: etiology, clinical findings, and consequences. J Steroid Biochem Mol Biol. (2015) 145:226–36. 10.1016/j.jsbmb.2014.06.004 24923732

[25] Reisch N Hogler W Parajes S Rose IT Dhir V Gotzinger J. A diagnosis not to be missed: nonclassic steroid 11beta-hydroxylase deficiency presenting with premature adrenarche and hirsutism. J Clin Endocrinol Metab. (2013) 98:E1620–5. 10.1210/jc.2013-1306 23940125

[26] Utriainen P Voutilainen R Jaaskelainen J. Continuum of phenotypes and sympathoadrenal function in premature adrenarche. Eur J Endocrinol. (2009) 160:657–65. 10.1530/EJE-08-0367 19151133

[27] Utriainen P Laakso S Liimatta J Jaaskelainen J Voutilainen R. Premature adrenarche–a common condition with variable presentation. Horm Res Paediatr. (2015) 83:221–31. 10.1159/000369458 25676474

[28] Pignatelli D. Non-classic adrenal hyperplasia due to the deficiency of 21-hydroxylase and its relation to polycystic ovarian syndrome. Front Horm Res. (2013) 40:158–70. 10.1159/000342179 24002412

[29] Skordis N Shammas C Efstathiou E Kaffe K Neocleous V Phylactou LA. Endocrine profile and phenotype-genotype correlation in unrelated patients with non-classical congenital adrenal hyperplasia. Clin Biochem. (2011) 44:959–63. 10.1016/j.clinbiochem.2011.05.013 21635882

[30] Conway G Dewailly D Diamanti-Kandarakis E Escobar-Morreale HF Franks S Gambineri A. The polycystic ovary syndrome: a position statement from the European Society of Endocrinology. Eur J Endocrinol. (2014) 171:P1–29. 10.1530/EJE-14-0253 24849517

[31] Honour JW. 17-Hydroxyprogesterone in children, adolescents and adults. Ann Clin Biochem. (2014) 51:424–40. 10.1177/0004563214529748 24711560

[32] Rosenfield RL Mortensen M Wroblewski K Littlejohn E Ehrmann DA. Determination of the source of androgen excess in functionally atypical polycystic ovary syndrome by a short dexamethasone androgen-suppression test and a low-dose ACTH test. Hum Reprod. (2011) 26:3138–46. 10.1093/humrep/der291 21908468 PMC3196876

[33] Holmes-Walker DJ Conway GS Honour JW Rumsby G Jacobs HS. Menstrual disturbance and hypersecretion of progesterone in women with congenital adrenal hyperplasia due to 21-hydroxylase deficiency. Clin Endocrinol. (1995) 43:291–6. 10.1111/j.1365-2265.1995.tb02034.x 7586597

[34] Auchus RJ Arlt W. Approach to the patient: the adult with congenital adrenal hyperplasia. J Clin Endocrinol Metab. (2013) 98:2645–55. 10.1210/jc.2013-1440 23837188 PMC3701266

[35] Charmandari E Brook CG Hindmarsh PC. Classic congenital adrenal hyperplasia and puberty. Eur J Endocrinol. (2004) 151(Suppl. 3):U77–82. 10.1530/eje.0.151u077 15554890

[36] Deslauriers JR Lenz AM Root AW Diamond FB Bercu BB. Gender related differences in glucocorticoid therapy and growth outcomes among pubertal children with 21-hydroxylase deficiency congenital adrenal hyperplasia (CAH). J Pediatr Endocrinol Metab. (2012) 25:977–81. 10.1515/jpem-2012-0125 23426829

[37] Hindmarsh PC Charmandari E. Variation in absorption and half-life of hydrocortisone influence plasma cortisol concentrations. Clin Endocrinol. (2015) 82:557–61. 10.1111/cen.12653 25369980

[38] Porter J Blair J Ross RJ. Is physiological glucocorticoid replacement important in children? Arch Dis Child. (2017) 102:199–205. 10.1136/archdischild-2015-309538 27582458 PMC5284474

[39] Saygili F Oge A Yilmaz C. Hyperinsulinemia and insulin insensitivity in women with nonclassical congenital adrenal hyperplasia due to 21-hydroxylase deficiency: the relationship between serum leptin levels and chronic hyperinsulinemia. Horm Res. (2005) 63:270–4. 10.1159/000086363 15956788

[40] Subbarayan A Dattani MT Peters CJ Hindmarsh PC. Cardiovascular risk factors in children and adolescents with congenital adrenal hyperplasia due to 21-hydroxylase deficiency. Clin Endocrinol. (2014) 80:471–7. 10.1111/cen.12265 23751160 PMC4204515

[41] Carmina E Dewailly D Escobar-Morreale HF Kelestimur F Moran C Oberfield S. Non-classic congenital adrenal hyperplasia due to 21-hydroxylase deficiency revisited: an update with a special focus on adolescent and adult women. Hum Reprod Update. (2017) 23:580–99. 10.1093/humupd/dmx014 28582566

[42] Falhammar H Frisen L Hirschberg AL Norrby C Almqvist C Nordenskjold A. Increased cardiovascular and metabolic morbidity in patients with 21-hydroxylase deficiency: a Swedish Population-Based National Cohort Study. J Clin Endocrinol Metab. (2015) 100:3520–8. 10.1210/JC.2015-2093 26126207

[43] Azziz R Dewailly D Owerbach D. Clinical review 56: nonclassic adrenal hyperplasia: current concepts. J Clin Endocrinol Metab. (1994) 78:810–5. 10.1210/jcem.78.4.8157702 8157702

[44] Carmina E Pathogenesis and treatment of hirsutism in late-onset congenital adrenal hyperplasia. Reprod Med Rev. (1995) 4:179–87. 10.1017/S0962279900000569

[45] Kim MS Merke DP. Cardiovascular disease risk in adult women with congenital adrenal hyperplasia due to 21-hydroxylase deficiency. Semin Reprod Med. (2009) 27:316–21. 10.1055/s-0029-1225259 19530065 PMC4830727

[46] Kamrath C Hochberg Z Hartmann MF Remer T Wudy SA. Increased activation of the alternative “backdoor” pathway in patients with 21-hydroxylase deficiency: evidence from urinary steroid hormone analysis. J Clin Endocrinol Metab. (2012) 97:E367–75. 10.1210/jc.2011-1997 22170725

[47] Kim MS Ryabets-Lienhard A Dao-Tran A Mittelman SD Gilsanz V Schrager SM. Increased abdominal adiposity in adolescents and young adults with classical congenital adrenal hyperplasia due to 21-hydroxylase deficiency. J Clin Endocrinol Metab. (2015) 100:E1153–9. 10.1210/jc.2014-4033 26062016 PMC4524992

[48] Stikkelbroeck NM Oyen WJ van der Wilt GJ Hermus AR Otten BJ. Normal bone mineral density and lean body mass, but increased fat mass, in young adult patients with congenital adrenal hyperplasia. J Clin Endocrinol Metab. (2003) 88:1036–42. 10.1210/jc.2002-021074 12629082

[49] Cali AM Caprio S. Obesity in children and adolescents. J Clin Endocrinol Metab. (2008) 93:S31–6. 10.1210/jc.2008-1363 18987268 PMC2585761

[50] Improda N Barbieri F Ciccarelli GP Capalbo D Salerno M. Cardiovascular health in children and adolescents with congenital adrenal hyperplasia due to 21-hydroxilase deficiency. Front Endocrinol. (2019) 10:212. 10.3389/fendo.2019.00212 31031703 PMC6470198

[51] Abd El Dayem SM Anwar GM Salama H Kamel AF Emara N. Bone mineral density, bone turnover markers, lean mass, and fat mass in Egyptian children with congenital adrenal hyperplasia. Arch Med Sci. (2010) 6:104–10. 10.5114/aoms.2010.13516 22371729 PMC3278952

[52] Macut D Antic IB Bjekic-Macut J. Cardiovascular risk factors and events in women with androgen excess. J Endocrinol Invest. (2015) 38:295–301. 10.1007/s40618-014-0215-1 25432327

[53] Laughlin GA Goodell V Barrett-Connor E. Extremes of endogenous testosterone are associated with increased risk of incident coronary events in older women. J Clin Endocrinol Metab. (2010) 95:740–7. 10.1210/jc.2009-1693 19934360 PMC2840853

[54] Falhammar H Filipsson Nystrom H Wedell A Thoren M. Cardiovascular risk, metabolic profile, and body composition in adult males with congenital adrenal hyperplasia due to 21-hydroxylase deficiency. Eur J Endocrinol. (2011) 164:285–93. 10.1530/EJE-10-0877 21098686

[55] Bachelot A Grouthier V Courtillot C Dulon J Touraine P. Management of endocrine disease: congenital adrenal hyperplasia due to 21-hydroxylase deficiency: update on the management of adult patients and prenatal treatment. Eur J Endocrinol. (2017) 176:R167–81. 10.1530/EJE-16-0888 28115464

[56] Wang L Szklo M Folsom AR Cook NR Gapstur SM Ouyang P. Endogenous sex hormones, blood pressure change, and risk of hypertension in postmenopausal women: the Multi-Ethnic Study of Atherosclerosis. Atherosclerosis. (2012) 224:228–34. 10.1016/j.atherosclerosis.2012.07.005 22862963 PMC3428144

[57] Diamanti-Kandarakis E Papavassiliou AG Kandarakis SA Chrousos GP. Pathophysiology and types of dyslipidemia in PCOS. Trends Endocrinol Metab. (2007) 18:280–5. 10.1016/j.tem.2007.07.004 17692530

[58] Arlt W Willis DS Wild SH Krone N Doherty EJ Hahner S. Health status of adults with congenital adrenal hyperplasia: a cohort study of 203 patients. J Clin Endocrinol Metab. (2010) 95:5110–21. 10.1210/jc.2010-0917 20719839 PMC3066446

[59] Mancini T Kola B Mantero F Boscaro M Arnaldi G. High cardiovascular risk in patients with Cushing's syndrome according to 1999 WHO/ISH guidelines. Clin Endocrinol. (2004) 61:768–77. 10.1111/j.1365-2265.2004.02168.x 15579193

[60] Botero D Arango A Danon M Lifshitz F. Lipid profile in congenital adrenal hyperplasia. Metabolism. (2000) 49:790–3. 10.1053/meta.2000.6261 10877208

[61] Charmandari E Weise M Bornstein SR Eisenhofer G Keil MF Chrousos GP. Children with classic congenital adrenal hyperplasia have elevated serum leptin concentrations and insulin resistance: potential clinical implications. J Clin Endocrinol Metab. (2002) 87:2114–20. 10.1210/jcem.87.5.8456 11994350

[62] Volkl TM Simm D Dotsch J Rascher W Dorr HG. Altered 24-hour blood pressure profiles in children and adolescents with classical congenital adrenal hyperplasia due to 21-hydroxylase deficiency. J Clin Endocrinol Metab. (2006) 91:4888–95. 10.1210/jc.2006-1069 17003094

[63] Roche EF Charmandari E Dattani MT Hindmarsh PC. Blood pressure in children and adolescents with congenital adrenal hyperplasia (21-hydroxylase deficiency): a preliminary report. Clin Endocrinol. (2003) 58:589–96. 10.1046/j.1365-2265.2003.01757.x 12699440

[64] Maccabee-Ryaboy N Thomas W Kyllo J Lteif A Petryk A Gonzalez-Bolanos MT. Hypertension in children with congenital adrenal hyperplasia. Clin Endocrinol. (2016) 85:528–34. 10.1111/cen.13086 27105393

[65] Harrington J Pena AS Gent R Hirte C Couper J. Adolescents with congenital adrenal hyperplasia because of 21-hydroxylase deficiency have vascular dysfunction. Clin Endocrinol. (2012) 76:837–42. 10.1111/j.1365-2265.2011.04309.x 22145701

[66] Wasniewska M Balsamo A Valenzise M Manganaro A Faggioli G Bombaci S Increased large artery intima media thickness in adolescents with either classical or non-classical congenital adrenal hyperplasia. J Endocrinol Invest. (2013) 36:12–5. 10.1007/BF03346751 22189488

[67] Falhammar H Filipsson Nystrom H Wedell A Brismar K Thoren M. Bone mineral density, bone markers, and fractures in adult males with congenital adrenal hyperplasia. Eur J Endocrinol. (2013) 168:331–41. 10.1530/EJE-12-0865 23211577

[68] King JA Wisniewski AB Bankowski BJ Carson KA Zacur HA Migeon CJ. Long-term corticosteroid replacement and bone mineral density in adult women with classical congenital adrenal hyperplasia. J Clin Endocrinol Metab. (2006) 91:865–9. 10.1210/jc.2005-0745 16278269

[69] Ambroziak U Bednarczuk T Ginalska-Malinowska M Malunowicz EM Grzechocinska B Kaminski P Congenital adrenal hyperplasia due to 21-hydroxylase deficiency - management in adults. Endokrynol Pol. (2010) 61:142–55. 20205117

[70] Jaaskelainen J Voutilainen R. Bone mineral density in relation to glucocorticoid substitution therapy in adult patients with 21-hydroxylase deficiency. Clin Endocrinol. (1996) 45:707–13. 10.1046/j.1365-2265.1996.8620871.x 9039336

[71] Falhammar H Filipsson H Holmdahl G Janson PO Nordenskjold A Hagenfeldt K. Fractures and bone mineral density in adult women with 21-hydroxylase deficiency. J Clin Endocrinol Metab. (2007) 92:4643–9. 10.1210/jc.2007-0744 17878254

[72] Paganini C Radetti G Livieri C Braga V Migliavacca D Adami S. Height, bone mineral density and bone markers in congenital adrenal hyperplasia. Horm Res. (2000) 54:164–8. 10.1159/000053253 11416232

[73] Ogilvie CM Rumsby G Kurzawinski T Conway GS. Outcome of bilateral adrenalectomy in congenital adrenal hyperplasia: one unit's experience. Eur J Endocrinol. (2006) 154:405–8. 10.1530/eje.1.02096 16498053

