# Supplementary material for: Metabolic Perspectives for Non-classical Congenital Adrenal Hyperplasia With Relation to the Classical Form of the Disease
Source: Front Endocrinol (Lausanne). 2019 Oct 2;10:681. doi: 10.3389/fendo.2019.00681 (PMC6783496; doi:10.3389/fendo.2019.00681)
Supplement: Supplementary file 1 [file Table_1.DOCX]

|  | **Non-classical CAH** | **Classical CAH** |
| --- | --- | --- |
| **Prevalence** | 1:1000 births (9) | 1:10000 – 1:20000 births (7,8) |
| **Clinical characteristics** | | |
| Growth | *Infancy and puberty*  Rapid linear growth and bone age advancement  may be present in boys and girls ([4](#_ENREF_4), [12](#_ENREF_12)).  Normal final height in most children (13)  Short stature on glucocorticoid therapy (13) | *Infancy and puberty*  SW boys and girls have birth length above the average but decline height velocity by age 1.5 in boys, and by age 3 in girls ([14](#_ENREF_14))  SV patients have absence of increased height velocity in the first year of life due to relative androgen insensitivity ([15](#_ENREF_15))  Pubertal growth spurt is not noticeable in both SW and SV form and genders ([16](#_ENREF_16))  *Adulthood*  SW and SV patients have reduced final adult height (18)  Women with SV CAH were significantly shorter than women with SW phenotype (18) |
| Sexual maturation | In cases with pubertal delay, development progress after initiation of glucocorticoid therapy with attainment of menarche and subsequently regular menstrual cycles ([22](#_ENREF_22)) | Onset of puberty for SV and SW girls at mean age of 9.8 and 10.3, respectively, and of menarche at mean age 13.3 and 13.7, respectively ([19](#_ENREF_19))  Onset of puberty for SV and SW boys at mean age of 9.8 and 10.6 years, respectively ([19](#_ENREF_19))  Earlier pubarche, gonadarche and thelarche in both sexes that is in contrast with the absence of typical adrenarche (20)  High adrenal androgens cause early puberty in both forms, more prevalent in SV than SW forms ([21](#_ENREF_21)) |
| **Therapy** | | |
| Glucocorticoids | Glucocorticoids for restoring fertility (34) | *Infancy and puberty*  Hydrocortisone dose adjustment for children to control growth and bone maturation (16)  Subcutaneous hydrocortisone infusion through a pump or modified-release hydrocortisone formulas are alternative options  to improve final height and metabolic outcomes ([38](#_ENREF_38))  Females at puberty need more hydrocortisone (37)  Longitudinal growth and bone age follow-up with frequent monitoring of 17-OHP, androstenedione and testosterone is requested in children on glucocorticoid replacement (35)  *Adulthood*  Prednisolone or dexamethasone for late puberty and adulthood (38)  *All ages*  Fludrocortisone for all patients with classical CAH ([13](#_ENREF_13)) |
| Antiandrogens and oral contraceptives | Antiandrogens and oral contraceptives for manifestations of androgen excess and changes in the menstrual cycle (34) |  |
| **Metabolic outcomes** | | |
| Obesity and cardiometabolic risk | *Infancy and puberty*  Children have high prevalence of obesity, hyperinsulinemia, hyperleptinemia (18)  Chronic androgen excess together with glucocorticoid administration around puberty favor abdominal visceral adiposity, insulin resistance and metabolic consequences ([45](#_ENREF_45))  *Adulthood*  Women have insulin resistance ([11](#_ENREF_11), [39](#_ENREF_39))  Males and females have increased risk of metabolic and cardiovascular morbidities ([42](#_ENREF_42)) | *Infancy and puberty*  Children, adolescents and young adults have high prevalence of obesity, hyperinsulinemia, hyperleptinemia (18, 47)  Increase in amount of fat commenced during childhood and even in children adequately treated ([48](#_ENREF_48))  *Adulthood*  Higher prevalence of obesity, hyperinsulinemia, hyperleptinemia and insulin resistance (18, [22](#_ENREF_22), 47)  Increase in amount of fat in young adults ([48](#_ENREF_48))  Life-long and higher doses of glucocorticoids are contributors to obesity and cardiovascular disease risk ([18](#_ENREF_18)) |
| Androgen excess and cardiovascular risk | Children has decreased HDL-C ([18](#_ENREF_18))  In younger children and adults hypertension is less prevalent (59)  Higher risk of having increased arterial intima-media thickness (63) | *Infancy and puberty*  Prepubertal children have elevated leptin and insulin levels (58), and elevated triglycerides when using prednisone ([57](#_ENREF_57))  Children has decreased HDL-C ([18](#_ENREF_18))  Hypertension is prevalent in younger children as a consequence of disease or as the effect of therapy (59)  *Adulthood*  Males older than 30 and adult women with androgen excess have increased cardiovascular risk including obesity, insulin resistance, dyslipidemia and cardiovascular outcomes as hypertension (18, 50, [52](#_ENREF_52), 55)  Atherogenic lipid profile with lower HDL-C and increased LDL-C levels ([54](#_ENREF_54))  Hypertension is prevalent in adults as a consequence of disease or as the effect of therapy (59)  Higher risk of having increased arterial intima-media thickness (63) |
| Bone mineral density (BMD) and risk for osteoporosis | *Adulthood*  Lower BMD in adult patients ([18](#_ENREF_18)) per se or in relation to glucocorticoid use ([64](#_ENREF_64))  Similar frequency of osteoporosis in comparison to classical CAH ([18](#_ENREF_18)) | *Adulthood*  Markers of bone formation as osteocalcin could be low in adult patients ([70](#_ENREF_70))  Adult patients with classical CAH are shorter in comparison to general population (17)  Lower BMD was demonstrated in adult patients on GCS (65, 66)  In women on GCS increased rate of osteoporotic fractures ([68](#_ENREF_68)) |

*Respective literature sources are given in brackets

NC-CAH, non-classical congenital adrenal hyperplasia; SW, salt waisting; SV, simple virilizing; PA, premature adrenarche; PCOS, polycystic ovary syndrome; ACTH, adrenocorticotropic hormone; 17-OHP, 17- Hydroxyprogesterone; HDL-C, high-density-lipoprotein cholesterol; BMD, bone mineral density; GCS, glucocorticosteroid.
